# Host Determinant Residue Lysine 627 Lies on the Surface of a Discrete, Folded Domain of Influenza Virus Polymerase PB2 Subunit

**DOI:** 10.1371/journal.ppat.1000136

**Published:** 2008-08-29

**Authors:** Franck Tarendeau, Thibaut Crepin, Delphine Guilligay, Rob W. H. Ruigrok, Stephen Cusack, Darren J. Hart

**Affiliations:** 1 Grenoble Outstation, European Molecular Biology Laboratory, Grenoble, France; 2 Unit of Virus Host-Cell Interactions, UJF-EMBL-CNRS, UMR5233, Grenoble, France; University of Wisconsin-Madison, United States of America

## Abstract

Understanding how avian influenza viruses adapt to human hosts is critical for the monitoring and prevention of future pandemics. Host specificity is determined by multiple sites in different viral proteins, and mutation of only a limited number of these sites can lead to inter-species transmission. Several of these sites have been identified in the viral polymerase, the best characterised being position 627 in the PB2 subunit. Efficient viral replication at the relatively low temperature of the human respiratory tract requires lysine 627 rather than the glutamic acid variant found systematically in avian viruses. However, the molecular mechanism by which any of these host specific sites determine host range are unknown, although adaptation to host factors is frequently evoked. We used ESPRIT, a library screening method, to identify a new PB2 domain that contains a high density of putative host specific sites, including residue 627. The X-ray structure of this domain (denoted the 627-domain) exhibits a novel fold with the side-chain of Lys627 solvent exposed. The structure of the K627E mutated domain shows no structural differences but the charge reversal disrupts a striking basic patch on the domain surface. Five other recently proposed host determining sites of PB2 are also located on the 627-domain surface. The structure of the complete C-terminal region of PB2 comprising the 627-domain and the previously identified NLS-domain, which binds the host nuclear import factor importin alpha, was also determined. The two domains are found to pack together with a largely hydrophilic interface. These data enable a three-dimensional mapping of approximately half of PB2 sites implicated in cross-species transfer onto a single structural unit. Their surface location is consistent with roles in interactions with other viral proteins or host factors. The identification and structural characterization of these well-defined PB2 domains will help design experiments to elucidate the effects of mutations on polymerase–host factor interactions.

## Author Summary

There is worldwide concern that currently circulating avian influenza viruses will cross the species barrier and become highly pathogenic, human transmissible strains with pandemic potential. This could result from residue changes in several influenza proteins, either by point mutations, or through shuffling of the segmented avian and mammalian viral genomes. Numerous studies have highlighted potentially important residues for inter-species transmission, and several are found in the influenza polymerase that replicates and transcribes the viral genome. The polymerase PB2 subunit contains a number of such positions, notably residue 627, which is glutamic acid in avian viruses but lysine in human-adapted strains. Experiments have shown that the polymerase mutations affect the efficiency of viral replication in different host species, but the molecular mechanisms are unknown. As a first step towards resolving this enigma, we have identified a novel domain of PB2, containing many host determinant sites, and determined its atomic structure by X-ray crystallography. The species-specific residues are all located on the domain surface, suggesting they could be involved in interactions with viral proteins or host factors. The 627 position is solvent-exposed in both the lysine and glutamic acid variants, respectively either reinforcing or disrupting a striking positively charged surface patch. The identification and structural characterisation of biochemically well-behaved domains of PB2 provides new tools for understanding the phenomenon of inter-species transmission that is of global health importance.

## Introduction

Influenza A viruses are orthomyxoviruses possessing an eight segment RNA genome of negative polarity. Each segment is packaged in a ribonucleoprotein complex (RNP) together with the nucleoprotein (NP) and the three subunits (PA, PB1 and PB2) of the trimeric RNA-dependent RNA polymerase, which mediates viral transcription and replication in the host cell nucleus [1]. Influenza A viruses with sixteen different sub-types of haemagglutinin (HA) endemically infect wild waterfowl, and frequently avian strains cause serious outbreaks of disease in domestic poultry. Influenza A viruses have also adapted to infect mammals and are a constant health risk for humans, causing seasonal epidemics and, more rarely, serious pandemics. The latter can arise when genome reassortments occur between avian and human strains [2] or when avian strains mutate to become infectious to and transmissible between humans, resulting in highly pathogenic viruses to which the human population is not immune [3]. This occurred notably in 1918 and led to an estimated 20–40 million deaths. Currently, highly pathogenic H5N1 avian strains are of worldwide concern because with only a few mutations they acquire the ability to infect humans with 60% mortality (http://www.who.int/csr/disease/avian_influenza/country/en/), although fortunately systematic human-to-human transmission of such strains has not yet been reported. It is therefore of great importance to understand the molecular mechanism of avian to human host adaptation, as well as the factors leading to high virulence, as this will contribute to effective monitoring of the likelihood of a pandemic, the development of new diagnostic tools and therapeutic strategies, and global counter-pandemic planning. Studies have identified specific features of the receptor binding glycoprotein, HA, the non-structural protein 1 (NS1) and the polymerase as being critical for both inter-species transmission and virulence [3]. Here we focus on the accumulating evidence that the viral polymerase plays a major role in avian to human transmission and that this is at least partly due to the requirement of the polymerase to adapt to interacting host factors [4]–[9].

The PB2 residue at position 627, which in nearly all human and avian influenza strains is either a lysine or glutamate respectively, is the best characterised polymerase determinant of host range and virulence. It was first identified using single gene reassortment viruses showing that the avian glutamic acid variant had restricted replication in mammalian cells and that a change to lysine restored viability [10]. Infection of mice with H5N1 influenza viruses from a 1997 human outbreak in Hong Kong was either lethal or non-lethal depending on the presence of Lys627 or Glu627, respectively [11]. Whereas viral replication of these two strains did not differ significantly in avian cell culture, growth in mouse cells exhibited a strong preference towards Lys627 [12]. Despite the clear importance of this residue for host specificity, little is known about the functional mechanism. One hypothesis is that residue 627 mediates interactions with essential host factors involved in RNA transcription and replication that differ between mammalian and avian species [13]–[15]. Although a number of polymerase-interacting proteins have been proposed as potential candidate host factors [9], [16]–[18], none have been specifically associated with the 627 position. A second hypothesis relates the nature of the 627 residue with the temperature optimum of viral replication. In humans, influenza viruses replicate in the upper respiratory tract at about 33°C whereas in birds replication occurs in the intestinal tract at 38–41°C. In an RNA replication assay with reconstituted RNPs, Lys627-containing polymerase replicated more efficiently in mammalian cells at 33°C than polymerase with Glu627, whereas at 37°C the difference was less marked [19]. Lys627 strains were subsequently shown to replicate more efficiently than Glu627 strains in the cooler lung and nasal turbinate tissues of mice, thus providing an environment for positive selection of this mutation [20].

In addition to residue 627, PB2 residues 701 and 714 are also implicated in host specific differences in polymerase efficiency, as revealed by laboratory studies of the adaptation of pathogenic avian strains to mice [5],[21],[22]. A study from our laboratory provided the first structural insight into these host determinant sites. We identified a C-terminal PB2 domain (residues 678–759) bearing a bipartite nuclear localisation sequence (NLS) via screening of a random library of expression constructs in *E. coli* [23]. The solution NMR structure of this domain (denoted NLS-domain) revealed the surface-exposed nature of Asp701 and Ser714 as well as Arg702, a naturally occurring host specific residue, which, with only rare exceptions, is an arginine in human isolates and a lysine in avian strains [4],[24],[25]. The X-ray co-crystal structure of the NLS-domain bound to the nuclear import factor human importin α5 provided an atomic-level insight of PB2 interacting with a host factor. A direct contact observed between Asp701 and the flexible NLS-containing C-terminus of PB2 suggested a role in modulating the PB2-importin interaction and nuclear import efficiency. Subsequently it was shown that the substitution D701N significantly affects the interaction of PB2 with importin α1 in mammalian but not avian cells [9].

More generally, statistical analysis of multiple sequence alignments based on large-scale influenza virus genome sequencing of avian and human isolates [26],[27] allows identification of candidate mutations that might contribute towards host specificity [24],[25],[28]. An extensive analysis of thousands of avian and human virus sequences identified 32 persistent host markers in 5 of the 11 viral proteins: PB2, PA, NP, M1 and NS1 [24]. Of these, 26 localize to the replication complex components PB2, PA and NP. Another recent analysis identified 17 sites within the PB2 subunit as putatively involved in avian to human adaptation [25]. These results strongly support the notion that adaptation of the replication complex to the host cell environment is a key event in inter-species transmission.

Unfortunately, with the exception of the NLS-domain [23] and most recently, the central cap-binding domain of PB2 [29], the lack of atomic resolution structural information on the polymerase precludes any detailed understanding of the functional role of individual candidate residues. Here we extend the structure-based exploration of host determinant residues through the identification by random construct screening of a new PB2 domain (residues 538–693) that contains position 627 and is thus denoted the 627-domain. The high resolution crystal structure of this domain from a human influenza A strain and of the K627E variant, shows that it has a novel fold with Lys/Glu627 exposed to the solvent. We have also determined the structure of the complete C-terminal region of PB2 (residues 538–759) which contains both the 627-domain and the NLS-domain. These structures enable seven out of a total 17 host specific sites on PB2 [25] to be mapped in three dimensions. The majority are surface-exposed residues with the potential to interact with either components of the polymerase complex, or with host factors.

## Results

### Identification of 627-NLS-domain soluble protein constructs

We identified two soluble protein constructs in the C-terminal region of PB2 from strain A/Victoria/3/1975(H3N2), via expression testing of random *pb2* gene fragments using the ESPRIT method [23],[29]. Fragment sizes of 150–250 amino acids were screened and several soluble constructs found that contained the NLS-domain together with an N-terminal extension beginning at residues 538 or 540. Minor proteolysis products observed during purification of the longer construct (538–759) led to the definition of two C-terminally truncated variants, comprising residues 538–693 (lacking the NLS-domain) and 538–753 (with a partly truncated NLS at the C-terminus of the NLS-domain). Both constructs yielded crystals diffracting to high resolution (1.1 Å and 1.9 Å respectively) from which their structures were determined. A notable feature of these protein fragments was the presence of Lys627. To assess the structural impact of the typically avian glutamate at this position, the K627E mutation was engineered into the 538–693 construct, the corresponding protein crystallized and its structure also determined.

### Structure of the 627-domain and location of position 627

**Figure 1:**
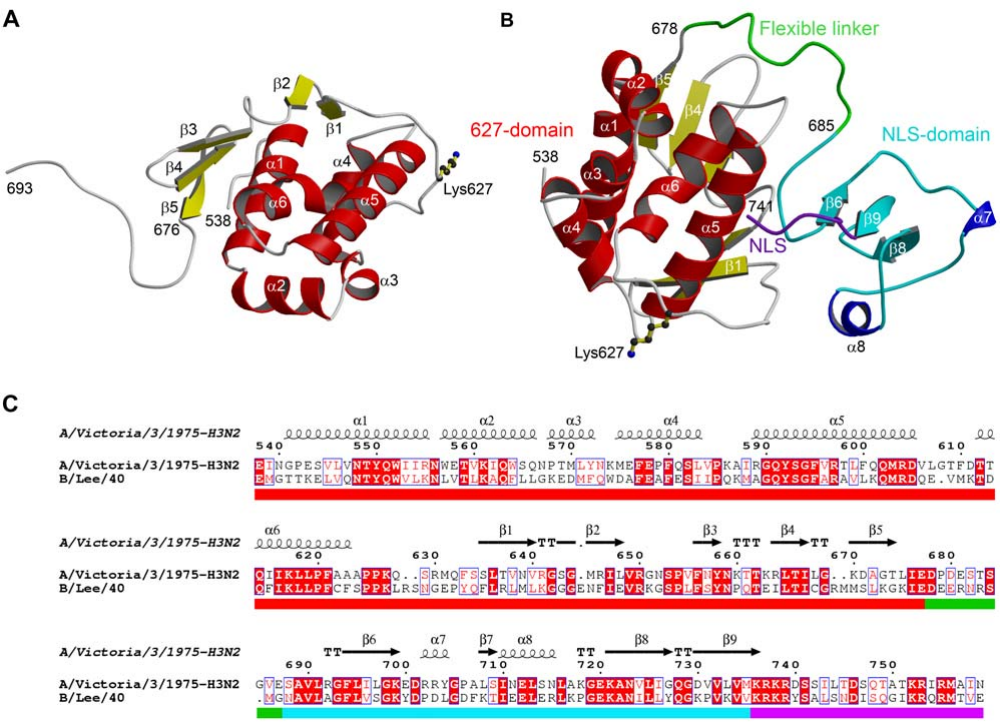
Structure and sequence alignment of C-terminal domains of influenza polymerase PB2 subunit. (A) Ribbon diagram of the 627-domain showing secondary structure elements and the position of human specific lysine 627. Helices are in red and beta-strands in yellow as defined by DSSP [30]. The conformation of the C-terminal tail (residues 676–693) is determined by crystal contacts. The structure shown is of the SeMet labelled protein. (B) Ribbon diagram of the 627-NLS-double domain showing the position of lysine 627. The 627-domain is in red and yellow, the core NLS-domain in cyan and blue and the truncated nuclear localization peptide in purple. The flexible inter-domain linker is in green. Figure 1A and Figure 1B were drawn with MOLSCRIPT [42] and rendered with RASTER3D [43]. (C) Sequence alignment of C-terminal regions of PB2 from influenza A and B viruses with superimposed secondary structure. The coloured bar under the alignment indicates the 627-domain (red), linker (green), core NLS-domain (cyan) and the bipartite NLS (purple). The seven host specific residues identified in this region [25] are indicated with a blue square in the coloured bar. Alignment figure produced with ESPript [44].

**Figure 2:**
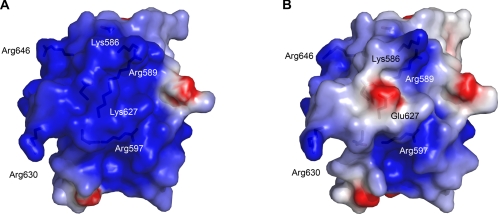
Effect of the K627E mutation on the electrostatic surface of the 627-domain. The electrostatic surface potentials were calculated from the crystal structures of (A) the Lys627 human determinant-containing domain, and (B) the Glu627 avian-like variant using DelPhi [45] and displayed using PyMol [46]. The potential scale ranges from -4 kT/e (red) to 4 kT/e (blue). The maps reveal that the K627E substitution disrupts a prominent basic surface patch which also includes residues Lys586, Arg589, Arg597, Arg630 and Arg646.

**Figure 3:**
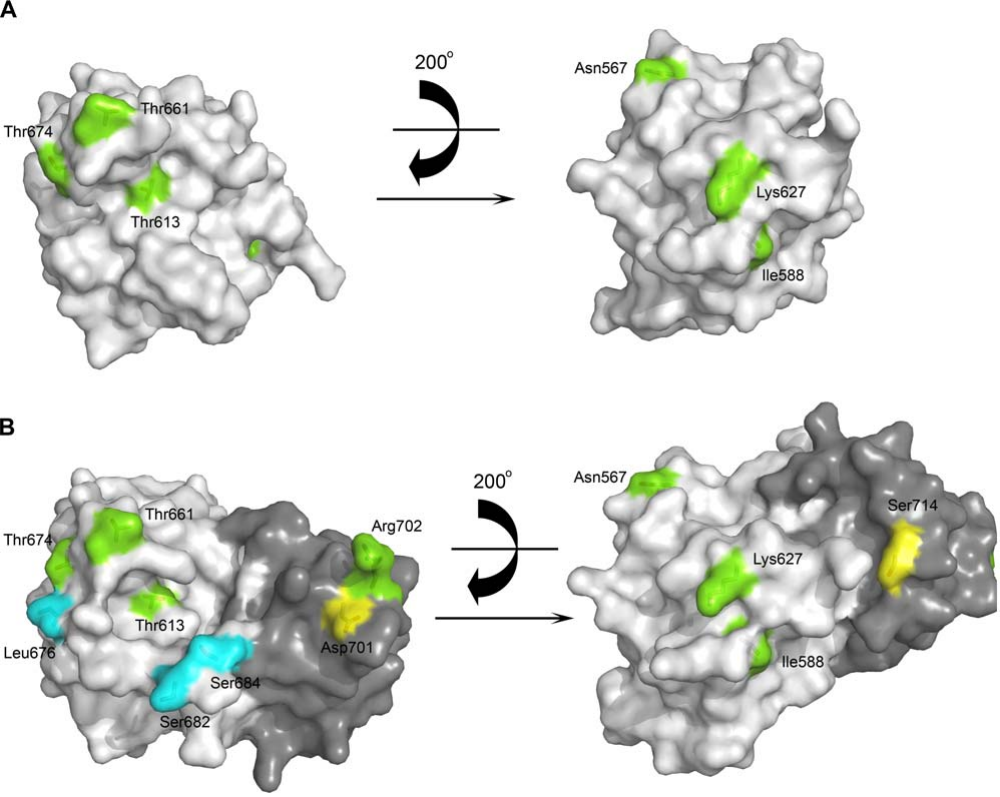
Identification of host species determinant sites. Surface representations highlight the position of the sites on the (A) 627-domain and (B) 627-NLS-domain in two different orientations. In (B) the 627-domain and NLS domain are respectively in grey and dark grey. Major host specificity determinant residues [25] are shown in green and with those for HxN2 subtype in blue. Residues 714 and 701 (yellow) were identified as host specificity determinants in a laboratory model of avian to mouse transmission [5].

PB2 residues 538–676 form a compact, highly ordered domain with a novel fold as indicated by the lack of structural homologues found by DALI [30] (Figure 1). The N-terminal half (residues 538–623) comprises a 6 helical cluster with a hydrophobic core rich in aromatic residues. Some of these aromatic residues, notably in the vicinity of Trp552 have previously been implicated in capped RNA binding by cross-linking studies [31],[32]. However the structure gives no indication that they would form a ligand binding site and furthermore it is now clear that the cap-binding site is located elsewhere in PB2 [29], although it cannot be ruled out that polymerase-bound mRNA also interacts with the 627-domain. The C-terminal half of the 627-domain (residues 635–676) comprises five short beta-strands which wrap around one side of the helical bundle (Figure 1A). Linking the alpha- and beta- halves of the domain is an extended peptide which wraps around helix α5 and contains the host-specific residue 627. This local region contains most of the residues strictly conserved between influenzas A, B and C (Figure 1C and Figure S4). The side-chain of Lys627 is fully solvent exposed and indeed electron density beyond Cγ is lacking. In the crystal, the C-terminal extension of the domain (residues 676–693) exhibits an extended conformation which is determined by the crystal packing. The structure of the K627E mutant domain is essentially identical (RMSD of 0.34 Å for all Cα atoms of residues 539–675), with the Glu627 side-chain (not visible beyond Cγ) again pointing into solvent. Thus the mutation induces neither local nor global changes in the domain structure. However, the charge reversal causes a major perturbation of the electrostatic surface of the domain (Figure 2). A number of other host determinant sites are also surface exposed on the 627-domain (Figure 3). The domain fold is unlike any other known protein so the structure in itself does not shed light on the functional role of residue 627, although its exposed surface location suggests it might mediate an interaction with another viral or host protein.

### Structure of the double 627-NLS-domain

The 1.95 Å resolution structure of the double 627-NLS-domain (residues 539–753) shows that the two domains pack side by side forming a single module (Figure 1B). The well-structured part of each domain shows only minor differences from that observed in either the isolated 627-domain (RMSD of 0.5 Å for all Cα atoms of residues 540–675) or the NLS-domain in complex with importin α5 [23] (RMSD of 0.78 Å for all Cα atoms of residues 694–738). Only the inter-domain linker (residues 678–692) and the visible part of the bipartite NLS (residues 736–741), both presumably flexible, show different conformations (Figure S1 and Figure S2). The inter-domain linker comprises two parts: residues 678–685 form a poorly ordered, flexible region while residues 686–692 form a well-ordered interface between the two domains. This interface comprises 11 hydrogen bonds including one salt bridge (Glu687 to Arg650) as well as burying hydrophobic residues on helix α5 of the 627-domain and on the NLS-domain (Figure S3). According to PISA (http://www.ebi.ac.uk/msd-srv/prot_int/pistart.html), the interface buries respectively 820 and 925 Å^2^ of solvent accessible surface of the 627- and NLS-domains. Given the flexible nature of the linkage between the two domains, it remains to be seen whether this moderately strong interface is of biological significance. The complete 627-NLS double domain (residues 538–759) was observed to form a stable complex *in vitro* with human importin α1 by size exclusion chromatography (Figure S5) as shown for the NLS-domain alone with human importin α5 [23]. A functional PB2-importin α1 interaction has previously been demonstrated by cellular studies [9].

## Discussion

The influenza polymerase has long resisted atomic resolution structural studies due to the problem of obtaining large amounts of material in soluble form. An important aspect of this is the inability to predict bioinformatically the domain structure of the polymerase subunits due to their unique sequences, apart from the polymerase domain of PB1. Previously we have used ESPRIT, a new method for screening for soluble protein fragments from random gene truncation libraries, to identify two functional domains from PB2; the C-terminal NLS-domain involved in nuclear import [23] and the cap-binding domain that participates in the ‘cap-snatching’ mode of transcription of viral mRNAs by binding the m^7^GTP 5′ extremity of host pre-mRNAs [29]. This domain-based approach has allowed us to derive the first high resolution structural information about this previously recalcitrant complex, although an understanding of how these domains function in the active trimeric complex clearly awaits a structure determination of the complete polymerase. Here, we have identified a third *E. coli* expressible domain from PB2 (538–693), termed the 627-domain after the most well-characterised host determinant site contained within it at position 627. The 156 amino acid 627-domain is located between the cap-binding and NLS-domains and contains six of the seventeen host species determining sites described within the PB2 subunit: N567D/E, I588A/V, T613V/A, K627E, T661A, T674A/S (Figure S4) where for each position the consensus human and then avian residues are given [25]. Thus the definition of this new domain locates a high density of host determinant sites onto a single structural unit (Figure 3). By contrast the similar sized PB2 cap-binding domain (residues 318–483)[29] has only two host determinant sites (K368R and M475L) [25]. The complete C-terminal region, comprising both the 627-domain and NLS-domain, also includes the host variable residue R702K and, in the inter-domain linker, the HxN2 subtype host determinants S682G and S684A [25].

The atomic structures reveal that all seven of the host-determining residues are located on the surface of the double domain (Figure 3). In addition, the residues 701 and 714, whose mutation (respectively D701N and S714R) have been shown to affect polymerase activity in a laboratory model of adaptation of virulent strains from birds to mice [5],[22], are located on the surface of the NLS-domain. The Lys627 side-chain is solvent exposed and forms part of a striking, positively-charged surface patch which also includes residues Lys586, Arg589, Arg597 and Arg630 (Figure 2A). This basic region is severely disrupted by the charge reversal upon mutation to glutamic acid (Figure 2B). In crystal structures of both the Lys and Glu variants of the 627-domain, superposition reveals no structural rearrangement of the domain upon mutation and in each case the side-chain is solvent exposed and partially disordered, most likely due to multiple conformations. The temperature effects on viral replication observed previously cannot therefore be explained in simple terms of structural differences between the Glu and Lys variants, at least at the domain level. Although, the role of the 627 amino acid remains enigmatic, the occurrence of a high density of host determinant residues on the surface of the C-terminal double domain of PB2 is suggestive that this region interacts with host factors, particularly in contrast to the PB2 cap-binding domain, which has a conserved intrinsic polymerase function, and a markedly low density of host determinant residues [25]. It is also possible that variant residues do not make direct protein contacts but, by affecting protein flexibility, help other regions to maintain polymerase activity or promote interactions with other domains or host factors.

From an analysis of H5N1 viruses isolated from infected humans in Vietnam it was observed that in 5/8 fatal and 3/4 non-fatal cases the E627K mutation had occurred [7]. Interestingly, in 3/4 cases retaining Glu627, but none of those with E627K, the D701N mutation was also found, leading to the suggestion that the latter mutation may compensate for the lack of change at position 627. Since we have hypothesised that position 701 may be involved in modulating the interaction with the nuclear import factor importin α [9],[23], we were prompted into investigating whether position 627 could also interact with the same host factor. Mixing purified 627-NLS-domain and importin α1 resulted in a stable complex as observed by size exclusion chromatography (Figure S5). A superposition of the double 627-NLS-domain structure, assuming it to be rigid, on that of the NLS-domain complexed with importin α5 via the common NLS-domain shows that there would be a significant clash of the 627-domain with the C-terminal region of importin α. Thus binding to importin α of the full-length PB2 (which includes the double domain), rather than just the extreme C-terminal NLS-domain, would require some flexibility at the level of the link between the bipartite NLS peptide and the core of the NLS-domain, if the double domain remains a rigid unit as observed crystallographically. Alternatively PB2 binding to importin α might only be possible if the 627- and NLS-domains were juxtaposed differently than observed in the crystal. Both possibilities are plausible, especially given the nature of the flexible linker between the domains and the fact that each domain is independently folded and soluble. Thus both the structure of the double domain in the context of the separate full-length PB2 subunit or the trimeric polymerase and the possible interaction of the 627-domain with importin α (or any other host factor) remain open questions.

It is reasonable to hypothesise that mutations of host determining sites in the influenza polymerase are required to adapt interactions with host specific factors. A number of putative host factors for the polymerase have been identified by two-hybrid [33],[34], proteomic [16],[18] and other methods [17], but with the exception of Asp701 and its possible influence on importin binding [9],[23], the effects of mutations upon interactions with these putative partners have not been investigated. The structurally compact and biochemically well-behaved PB2 domains characterised here and previously [23],[29] will facilitate improved screens using well-defined bait proteins and should result in more specific interactions being identified. The effects of mutations upon protein-protein affinities of these domain-host factor complexes can then be measured and structural studies will help in understanding the nature of the interaction interfaces and the contributions of the surface-exposed host determinant residues.

## Materials and Methods

### Identification of C-terminal expression constructs

The *pb2* gene from A/Victoria/3/1975(H3N2) was codon-optimised for expression in *E. coli* (Geneart) and cloned into a modified pET9a vector (Novagen) with N-terminal hexahistidine tag and TEV protease cleavage sequence (MGHHHHHHDYDIPTTENLYFQG) and C-terminal biotin acceptor peptide with linker (SNNGSGGGLNDIFEAQKIEWHE). Restriction site pairs 5′ *Aat*II/*Asc*I and 3′ *Nsi*I/*Not*I sites flanked the *pb2* gene enabling generation of internal gene fragments by sequential exonuclease III truncation reactions [23],[35] to generate a library of inserts fused to both tags after blunt-end generation and ligation [29]. Plasmids with 450-750 nucleotide *pb2* inserts were excised from 1% agarose gel prior to the second ligation step, then recovered by transforming Mach1 cells (Invitrogen). Purified DNA was prepared from about 35,000 pooled colonies and electroporated into *E. coli* strain BL21 AI (Invitrogen) containing the RIL plasmid (Stratagene). Colony blots of approximately 27,000 clones were hybridized with Alexa 488 streptavidin (Invitrogen) and screened for expression constructs by fluorimager [23]. Two clones expressing purifiable C-terminal PB2 proteins (amino acids 538–759 and 540–759) were identified within the first 96 most fluorescent clones. The 538–759 protein cleaved better with TEV protease, but exhibited some C-terminal proteolytic degradation at 4°C during storage. Mass spectrometry identification of these products revealed two stable fragments (538–693 and 538–753) that were sub-cloned in to the same modified pET9a vector as described above. The mutation K627E in the 538–693 protein was made by PCR mutagenesis.

### Protein purification and labelling

Native proteins were expressed in *E. coli* strain BL21 AI RIL in TB medium. Partially selenomethionine labelled 538–693 protein was produced using M9 medium supplemented with 50 mg/l of selenomethionine and 5 mg/l of methionine. Protein expression was induced by the addition of 0.2% w/v arabinose for 20 h at 25°C. Cells were resuspended and sonicated in lysis buffer (30 mM Tris-HCl pH 7.0, 200 mM NaCl). Proteins were purified on Ni^2+^ chelating sepharose column (GE Healthcare). Columns were intensively washed with 4 different buffers (30 mM Tris-HCl pH 7.0, 200 mM NaCl; 10 mM Tris-HCl pH 7.0, 1 M NaCl; 10 mM Tris-HCl pH 7.0, 200 mM NaCl, 50 mM imidazole; 10 mM Tris-HCl pH 7.0, 200 mM NaCl, 75 mM imidazole) and the proteins were eluted with elution buffer (10 mM Tris-HCl pH 7.0, 200 mM NaCl, 500 mM imidazole). The hexahistidine tag was removed with TEV protease overnight at 15°C leaving an additional N-terminal glycine residue. Proteins were dialyzed against 10 mM Tris-HCl pH 7.0, 200 mM NaCl and a second Ni^2+^ chelating sepharose column was used to remove unwanted material. Proteins were then purified by gel filtration on Superdex 75 column (GE Healthcare).

### Interaction assay between large C-terminal PB2 domain and human importin α1 by size exclusion chromatography

Hexahistidine-tagged human importin α1 (KPNA2; residues 60–529) and the 627-NLS-domain (with two additional C-terminal alanine residues from cloning) were purified and the affinity tags removed by TEV digestion. They were then mixed at a 1∶2 molar ratio (importin:PB2) overnight at 4°C. Proteins were concentrated to 5.5 mg/ml and purified using a Superdex S200 size exclusion column (GE Healthcare) in 10 mM Tris-HCl pH 7.0, 200 mM NaCl.

### Crystallization

Hanging drop vapour diffusion trials were performed at 20°C. Native, mutated and partially selenomethionine labelled 538–693 PB2 protein crystals were grown by mixing 1 µl of 2.4 mg/ml protein solution in 10 mM Tris-HCl pH 7.0 and 200 mM NaCl with 1 µl of 100 mM citric acid pH 4.0–7.0 and 1.4–1.6 M ammonium sulfate solution. Native 538–753 PB2 protein crystals were grown by mixing 1 µl of 5.5 mg/ml protein solution in 10 mM Tris-HCl pH 7.0 and 200 mM NaCl with equal volume of 100 mM Hepes pH 7.5 and K/Na Tartrate 1.2 M. Crystals were frozen in liquid nitrogen after soaking in crystallization solution supplemented with 30% glycerol.

### Crystallography

Crystals of the 627-domain (residues 538–693) with Lys627 (native and selenomethionine labelled), the 627-domain with Glu627 (native) and the 627-NLS-domain (native, residues 538–753) were measured at the European Synchrotron Radiation Facility (ESRF). Table 1 gives all data collection and refinement statistics. All crystals have one molecule in the asymmetric unit. All data were integrated with XDS [36] and analysed using the CCP4i package [37]. The structure of the 627-domain with Lys627 was solved by the SAD method using AUTOSHARP [38] which found 5 selenium positions. ARP/wARP [39] was used for automatic model building. The structure of the K627E mutant was obtained by refinement. The double domain structure was solved by molecular replacement using PHASER [40] and, as search models, the 627-domain and the NLS-domain from the complex with human importin α5 (PDB id: 2JDQ). All refinements were performed with REFMAC [41] with added hydrogen atoms. For the very high resolution native 627-domain and K627E structures individual atomic anisotropic B-factors were refined. In the 627-domain alone structures, the 640–644 loop is disordered and the 609–610 loop has multiple conformations; both regions are well ordered in the 627-NLS-domain structure. The linker region 678–685 is poorly ordered in the 627-NLS-domain structure, but residual discontinuous density unambiguously defines which 627-domain is connected to which NLS-domain in the crystallographic asymmetric unit. Diffraction data for the 627-domain alone extend to very high resolution (1.1 Å for the native Lys627 data). Paradoxically the highest resolution data does not yield the most complete model; for example in the Lys627 native structure there is no electron density for the extended C-terminal tail of the 627-domain (residues 676–693), whereas this is perfectly ordered in the SeMet data and mostly ordered in the Glu627 data. In the latter two structures many multiple conformations can be modelled (Table 1). According to MOLPROBITY all structures have excellent geometry (http://molprobity.biochem.duke.edu/).

**Table 1:** Data collection and refinement statistics of the 627-domain (537–693) and the 627-NLS-domain (537–753).

	627-domain K627 native	627-domain K627 selenomethionine Peak wavelength	627-domain E627 native	627-NLS-domain native
**Data collection**				
Beamline (ESRF)	BM14	BM14	ID23-2	ID23-1
Wavelength (Å)	1.008	0.979	0.873	0.954
Space group	*C*2	*C*2	*C*2	*P*2_1_
Cell dimensions				
* a*, *b*, *c* (Å)	48.3, 45.5, 59.9	48.4, 45.0, 60.0	48.5, 45.7, 60.8	33.4, 65.4, 45.5
α, β, γ (°)	90.0, 105.4, 90.0	90.0, 105.5, 90.0	90.0, 106.2, 90.0	90.0, 92.0, 90.0
Resolution (Å)	50-1.10 (1.10–1.16)*	58–1.53 (1.53–1.58)*	30–1.15 (1.15–1.20)*	30–1.95 (1.95–2.00)*
*R*_merge_	0.031 (0.393)	0.042 (0.170)	0.098 (0.327)	0.128 (0.572)
*I*/σ*I*	16.8 (3.1)	20.2 (5.3)	5.07 (2.50)	8.95 (2.28)
Completeness (%)	94.4 (87.6)	97.6 (77.3)	92.9 (85.9)	98.3 (92.9)
Redundancy	3.66 (2.72)	3.67 (2.28)	2.84 (1.39)	4.01 (3.09)
**Refinement**				
Resolution (Å)	30–1.1 (1.10–1.13)*	58–1.53 (1.53–1.57)*	30–1.20 (1.20–1.23)*	30–1.95 (1.95–2.00)*
Total No. reflections/free	45464/2439	17685/960	35705/1874	13456/712
*R*_work_	0.181 (0.239)	0.144 (0.159)	0.189 (0.254)	0.200 (0.265)
*R*_free_	0.192 (0.266)	0.185 (0.251)	0.214 (0.281)	0.258 (0.390)
No. atoms	1212	1535	1404	1649
Protein	1083	1361	1279	1572
Water	129	174	125	77
No. residues modelled with multiple conformations	-	22	19	-
Average B-factors (Å^2^)	12.65	7.51	9.91	21.4
R.m.s. deviations				
Bond lengths (Å)	0.008	0.015	0.014	0.015
Bond angles (°)	1.26	1.57	1.54	1.61
Ramachandran Plot**				
Favoured (%)	100	97.9	100	98
Allowed (%)	100	100	100	100

*: Values in parentheses are for highest-resolution shell.

**: Molprobity: http://molprobity.biochem.duke.edu/

### Database deposition

The co-ordinates and structure factors of the PB2 domains are available from the PDB with codes 2vy6 for the 627-NLS double domain (native data), 2vy7 for the 627-domain with Lys627 (selenomethionine labelled protein) and 2vy8 for the 627-domain with Glu627 (native data).

## Supporting Information

Figure S1 Comparison of the structure of the isolated 627-domain (pink) with that in the double 627-NLS-domain (red). The domain is in the same orientation as that of Figure 1B. Helices are marked according to the secondary structure assignment. Significant differences are observed only in the conformation of the flexible region 676-693.
(4.30 MB TIF)

Figure S2 Comparison of the structures of the NLS-domain. The NLS-domain (blue) from the double 627-NLS-domain has been superimposed on that in the complex with human importin α5 (PDB: 2JDQ; light blue). Visible secondary structural elements are labelled as in the double 627-NLS-domain. Significant differences are observed at the two extremities of the domain. In particular residues 686-692 are helical in the importin complex but extended in the double domain.
(2.34 MB TIF)

Figure S3 Diagram showing hydrogen bonds at the interface between the 627- (red) and NLS- (cyan) domains in the 627-NLS-domain structure. The interface comprises 11 hydrogen bonds (dotted green with distances between acceptor and donor marked) including one salt bridge (Arg650 to Glu687). Several hydrophobic residues on helix α5 of the 627-domain (e.g. Phe595, Leu599, Met603, Val606) and from the NLS-domain (e.g. Ile710 and Ile726) are buried or partially buried at the interface. For clarity, these residues are not shown.
(2.94 MB TIF)

Figure S4 Sequence alignment of the 627-domain from human (H3N2) and avian (H5N1) strains of influenza A, influenza B and influenza C with superposed secondary structure. Residues with a red background are conserved in all strains; these are primariliy in the helices α5 and α6 in proximity to the residue 627, which is a lysine in all strains except avian. The purple boxes indicate differences between the human and avian influenza A strains. All the differences highlighted by Miotto et al. [25] occur as well as some non-consensus changes (G590C and I676T).
(0.52 MB TIF)

Figure S5 Interaction assay between large C-terminal PB2 domain and human importin α1 by size exclusion chromatography. Fractions were analyzed by SDS-PAGE revealing a major peak comprising a complex of importin α1 and 627-NLS-domain (fractions 25 to 29) and a minor peak containing excess unbound 627-NLS-domain (fractions 31 to 36). The 627-NLS domain alone eluted in fractions 31 to 36 (not shown).
(3.81 MB TIF)
