# Supplementary figures and images for: Host Determinant Residue Lysine 627 Lies on the Surface of a Discrete, Folded Domain of Influenza Virus Polymerase PB2 Subunit
Source: PLoS Pathog. 2008 Aug 29;4(8):e1000136. doi: 10.1371/journal.ppat.1000136 (PMC2515345; doi:10.1371/journal.ppat.1000136)

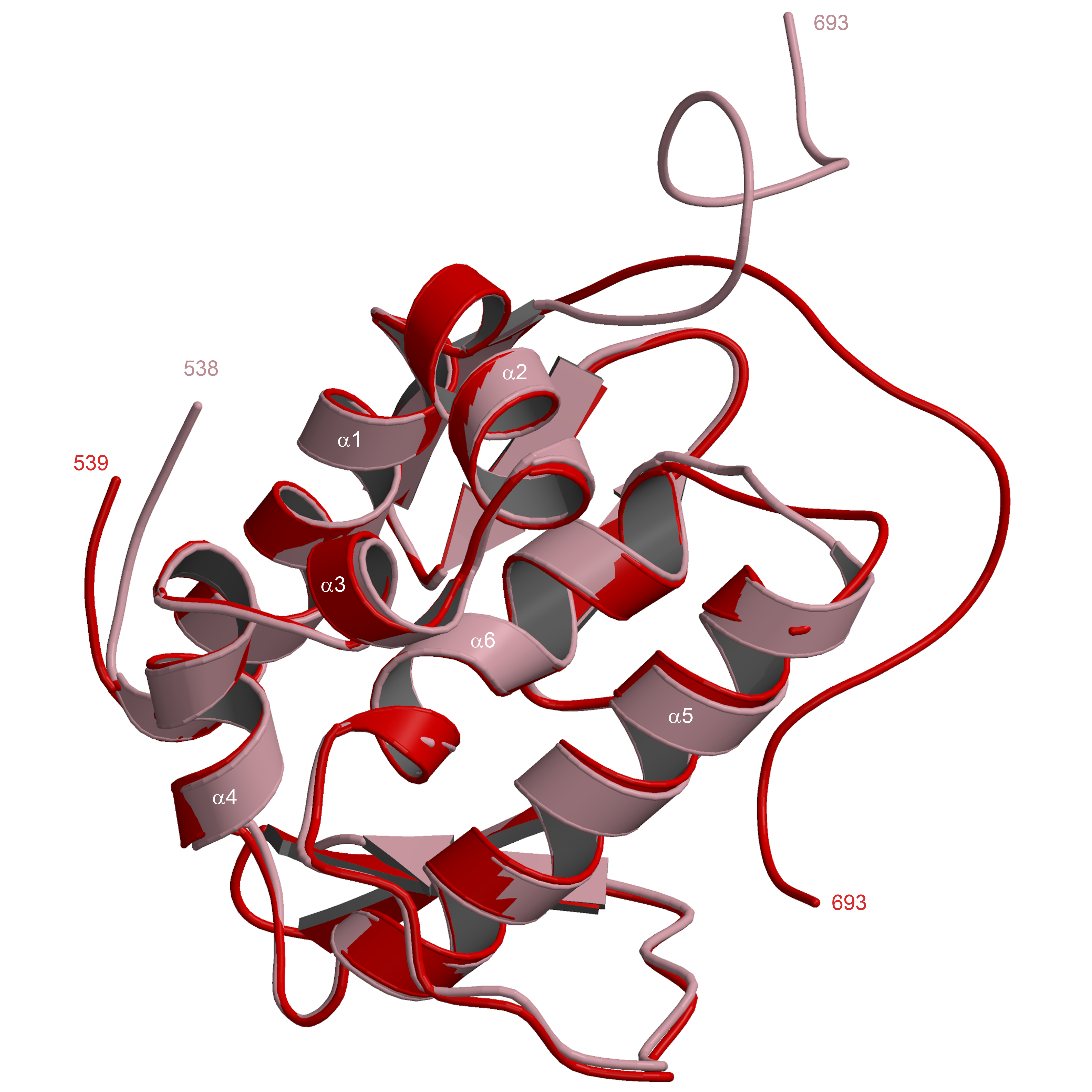

Supplement: Figure S1 — Comparison of the structure of the isolated 627-domain (pink) with that in the double 627-NLS-domain (red). The domain is in the same orientation as that of Figure 1B. Helices are marked according to the secondary structure assignment. Significant differences are observed only in the conformation of the flexible region 676-693. (4.30 MB TIF) [file ppat.1000136.s001.tif]

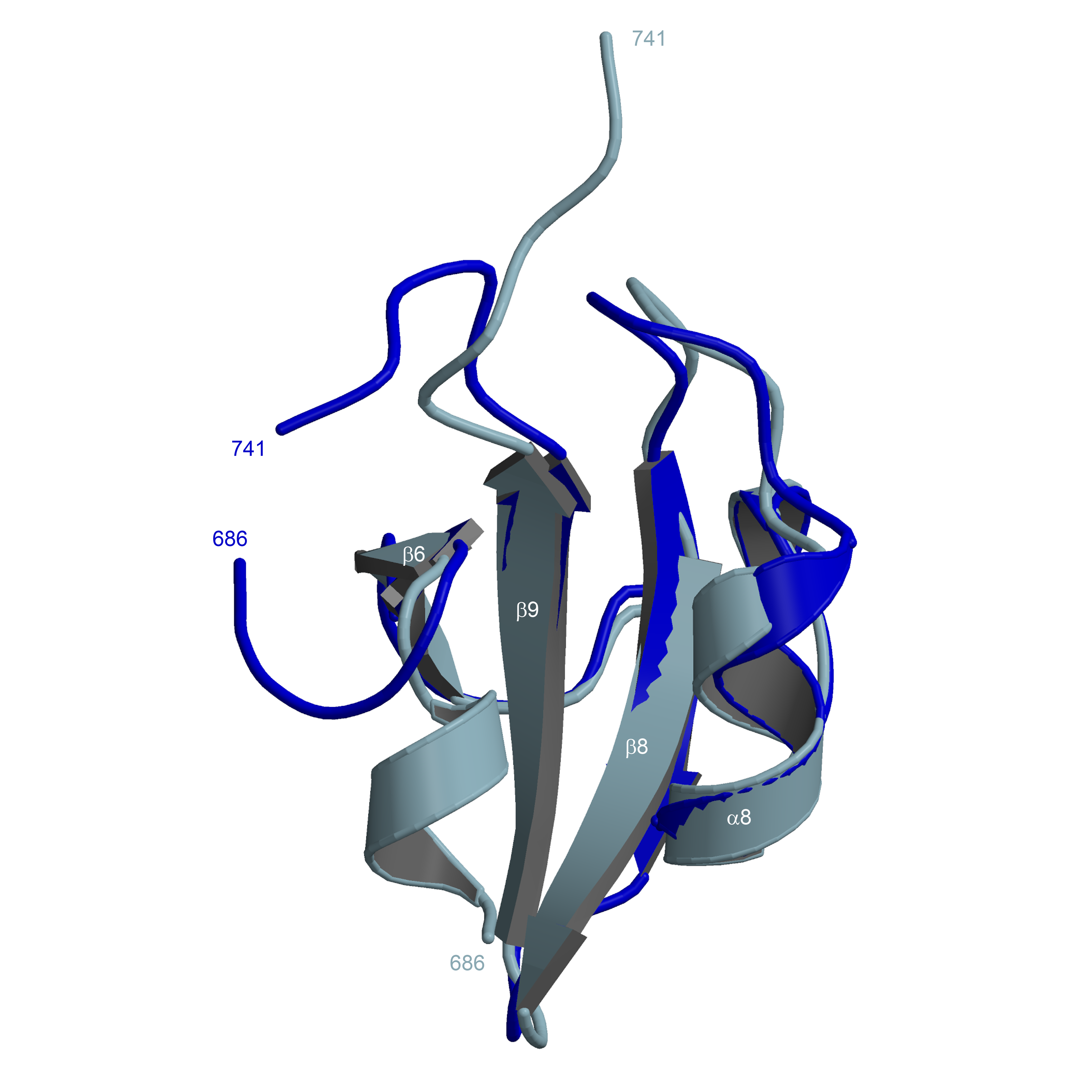

Supplement: Figure S2 — Comparison of the structures of the NLS-domain. The NLS-domain (blue) from the double 627-NLS-domain has been superimposed on that in the complex with human importin α5 (PDB: 2JDQ; light blue). Visible secondary structural elements are labelled as in the double 627-NLS-domain. Significant differences are observed at the two extremities of the domain. In particular residues 686-692 are helical in the importin complex but extended in the double domain. (2.34 MB TIF) [file ppat.1000136.s002.tif]

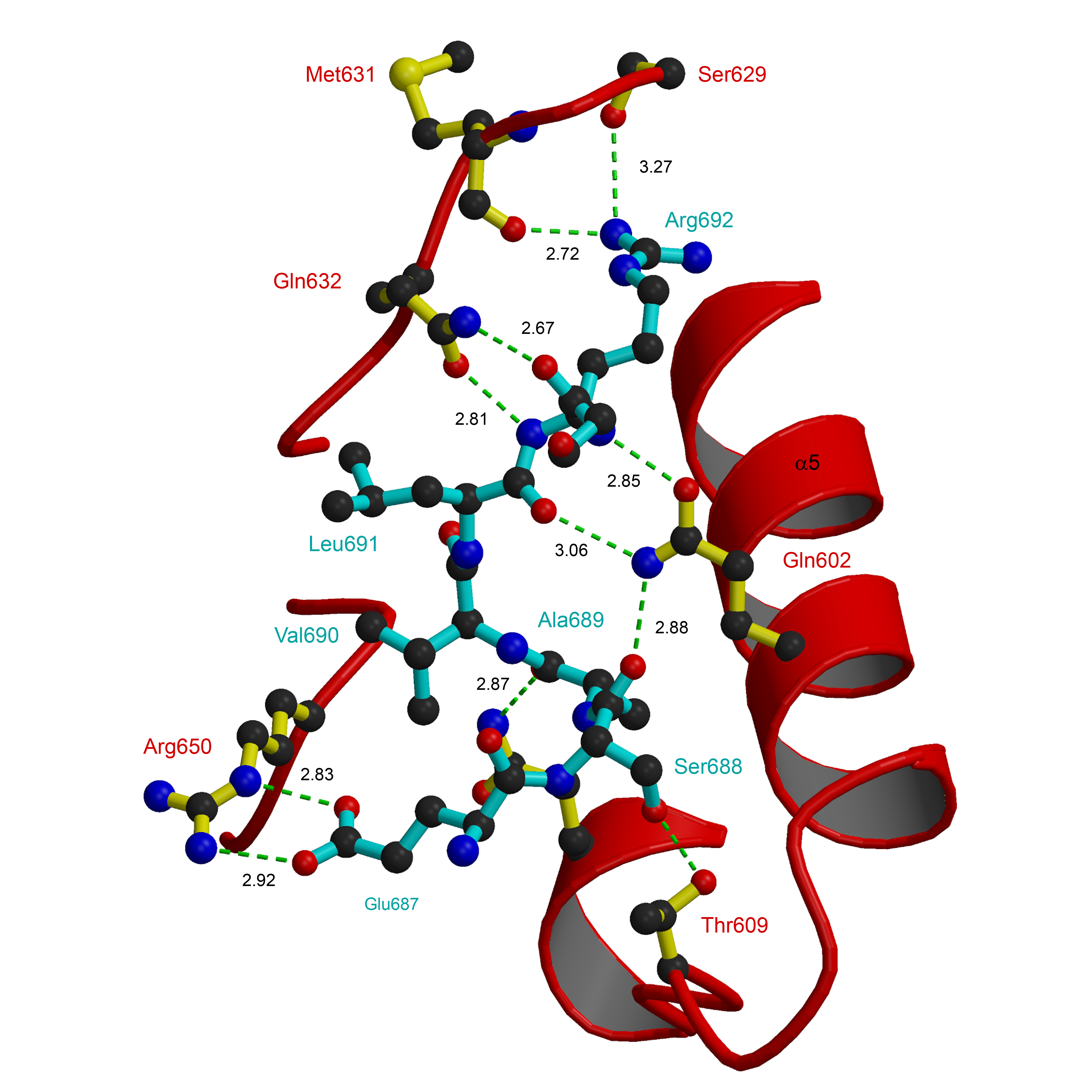

Supplement: Figure S3 — Diagram showing hydrogen bonds at the interface between the 627- (red) and NLS- (cyan) domains in the 627-NLS-domain structure. The interface comprises 11 hydrogen bonds (dotted green with distances between acceptor and donor marked) including one salt bridge (Arg650 to Glu687). Several hydrophobic residues on helix α5 of the 627-domain (e.g. Phe595, Leu599, Met603, Val606) and from the NLS-domain (e.g. Ile710 and Ile726) are buried or partially buried at the interface. For clarity, these residues are not shown. (2.94 MB TIF) [file ppat.1000136.s003.tif]

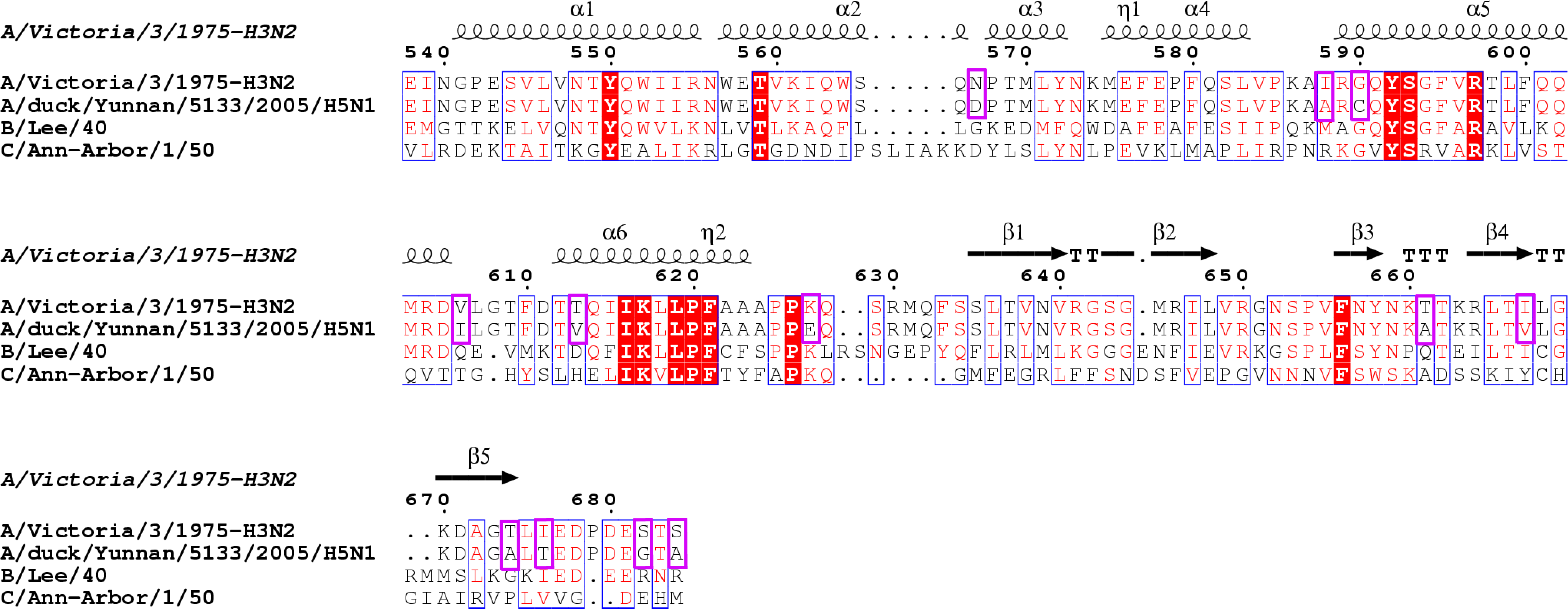

Supplement: Figure S4 — Sequence alignment of the 627-domain from human (H3N2) and avian (H5N1) strains of influenza A, influenza B and influenza C with superposed secondary structure. Residues with a red background are conserved in all strains; these are primariliy in the helices α5 and α6 in proximity to the residue 627, which is a lysine in all strains except avian. The purple boxes indicate differences between the human and avian influenza A strains. All the differences highlighted by Miotto et al. [25] occur as well as some non-consensus changes (G590C and I676T). (0.52 MB TIF) [file ppat.1000136.s004.tif]

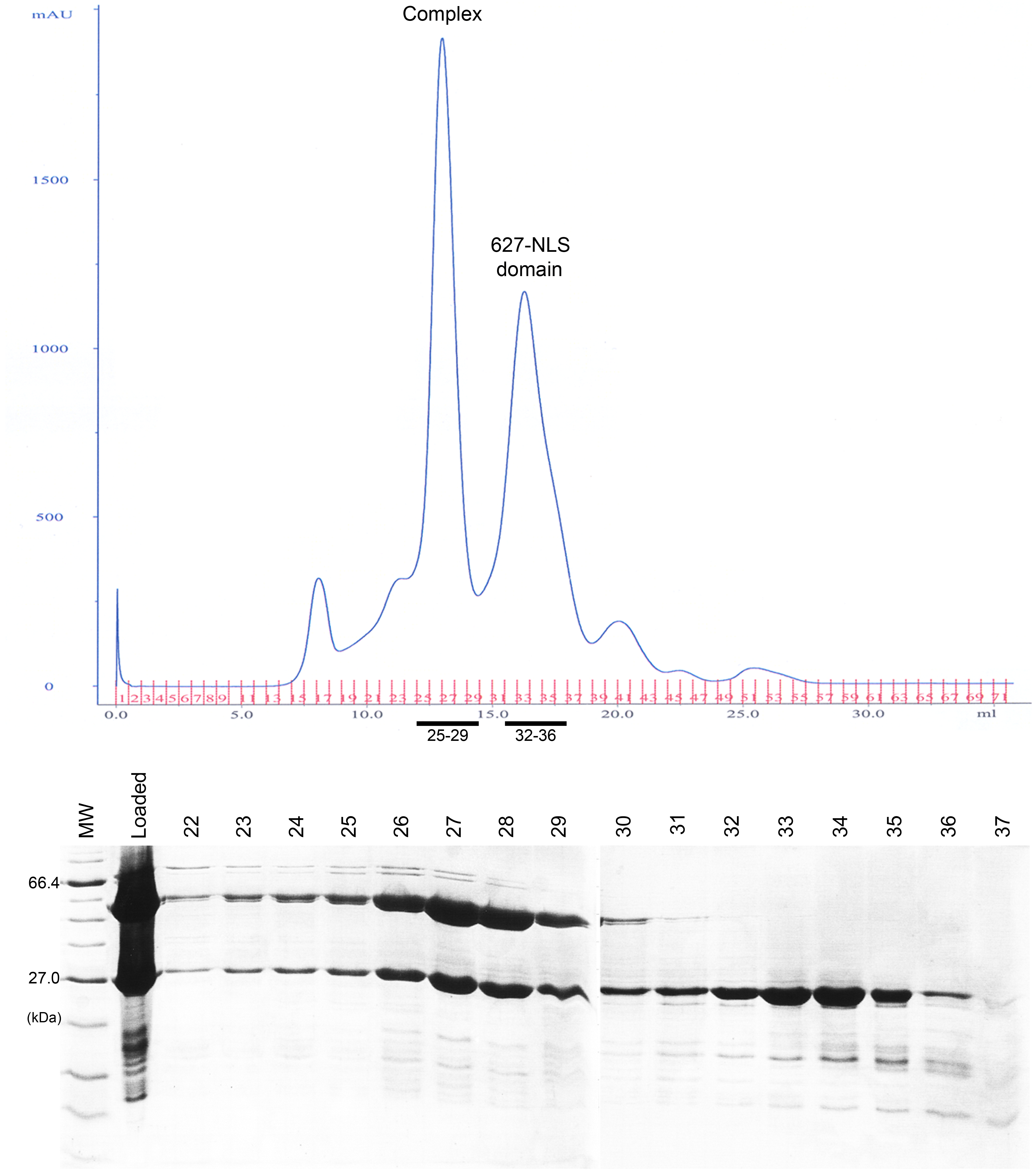

Supplement: Figure S5 — Interaction assay between large C-terminal PB2 domain and human importin α1 by size exclusion chromatography. Fractions were analyzed by SDS-PAGE revealing a major peak comprising a complex of importin α1 and 627-NLS-domain (fractions 25 to 29) and a minor peak containing excess unbound 627-NLS-domain (fractions 31 to 36). The 627-NLS domain alone eluted in fractions 31 to 36 (not shown). (3.81 MB TIF) [file ppat.1000136.s005.tif]
